# A device for single leaf labelling with CO_2_ isotopes to study carbon allocation and partitioning in *Arabidopsis thaliana*

**DOI:** 10.1186/1746-4811-9-45

**Published:** 2013-11-19

**Authors:** Katharina Kölling, Antonia Müller, Patrick Flütsch, Samuel C Zeeman

**Affiliations:** 1 Department of Biology, Institute of Agricultural Sciences, ETH Zurich, Universitätsstrasse 2, 8092 Zurich, Switzerland

**Keywords:** Arabidopsis, Photosynthesis, Carbohydrate metabolism, Assimilate partitioning, Phloem transport, Isotope labelling, Sink-source relationships

## Abstract

**Background:**

Plant biomass consists primarily of carbohydrates derived from photosynthesis. Monitoring the assimilation of carbon via the Calvin-Benson cycle and its subsequent utilisation is fundamental to understanding plant growth. The use of stable and radioactive carbon isotopes, supplied to plants as CO_2_, allows the measurement of fluxes through the intermediates of primary photosynthetic metabolism, long-distance transport of sugars in the vasculature, and the synthesis of structural and storage components.

**Results:**

Here we describe the design of a system for supplying isotopically labelled CO_2_ to single leaves of *Arabidopsis thaliana*. We demonstrate that the system works well using short pulses of ^14^CO_2_ and that it can be used to produce robust qualitative and quantitative data about carbon export from source leaves to the sink tissues, such as the developing leaves and the roots. Time course experiments show the dynamics of carbon partitioning between storage as starch, local production of biomass, and export of carbon to sink tissues.

**Conclusion:**

This isotope labelling method is relatively simple to establish and inexpensive to perform. Our use of ^14^CO_2_ helps establish the temporal and spatial allocation of assimilated carbon during plant growth, delivering data complementary to those obtained in recent studies using ^13^CO_2_ and MS-based metabolomics techniques. However, we emphasise that this labelling device could also be used effectively in combination with ^13^CO_2_ and MS-based techniques.

## Background

Central carbon metabolism has been studied for many decades with the help of both radioactive (^11^C and ^14^C) and stable (^13^C) carbon isotopes. Each isotope offers advantages and disadvantages. The ^11^C isotope has a very short half-life of 20.334 minutes but emits high energy positrons upon decay. Therefore, it is well suited for *in vivo* studies of phloem transport, partitioning of carbon between alternative sinks, such as roots, leaves and seeds [1,2], and to calculate the rates of carbon transport and leakage [3]. Furthermore, the short half-life means that the same biological material can be labelled sequentially (e.g. before and after a treatment; [4,5]). However, due to its short-lived nature and high energy emissions, ^11^C has major drawbacks. It has to be produced at the site of the experiment, it is difficult to handle, and it is expensive. The long-lived radioactive ^14^C (half-life of 5,700 years), on the other hand, is relatively cheap and easy to use for labelling experiments. Labelled plant material can be fractionated and its low-energy beta particles measured using scintillation counters. This allows carbon allocation into different tissues and carbon partitioning into metabolic pools to be accurately quantified. Radiolabeled material is not usually analysed by mass spectrometry (MS), so measurement of carbon partitioning into specific metabolites is laborious. However, ^13^C labelling is highly amenable with techniques such as MS and nuclear magnetic resonance (NMR), which allow metabolite identification and can provide positional information for the isotope label [6,7]. Furthermore, elemental analysis of ^13^C labelled material allows measurements of carbon allocation towards sink tissues to be made [8-10].

Isotope labelling has been pivotal to some of the groundbreaking work that elucidated the pathways of the Calvin-Benson cycle and photorespiration [11,12]. Sucrose and starch were shown to be the main products of photoassimilation [13] and sucrose was identified as the major form in which carbon is transported to sink tissues such as seeds, flowers and roots [14]. Experiments on sugar beet and tobacco showed that young leaves are supported by mature ones with carbon reserves until they reach 30-60% of their final length, whereupon they themselves turn from sink into source leaves [15-17]. The movement of carbon towards the sink tissues follows a defined pattern, determined by the vascular connections [18,19].

Recently, ^13^C labelling has been used to analyse central carbon metabolism. In metabolic flux analyses, heterotrophic or mixothrophic cells are fed with positionally ^13^C labelled sugars (e.g. [6-^13^C]-glucose) until steady state is reached. Determination of the isotope pattern in several pathway-relevant metabolites allows metabolic fluxes to be calculated, based on an existing metabolic model [20-22]. Metabolic fluxes have also been measured in autotrophic tissues, though this is more difficult as ^13^C has to be supplied in the form of gaseous ^13^CO_2_. In this case, reaching a steady state is not informative, as all carbon atoms in a metabolite become labelled and flux calculations would be impossible. Therefore, time-course experiments in a non-stationary state are performed [23]. Thus far, only a limited number of metabolites and their isotope patterns can be measured. Nevertheless, ^13^CO_2_ labelling will certainly gain in importance in the future, and good tools for labelling will be necessary.

Labelling with ^14^CO_2_ has been used extensively in the last decades to study the allocation of photo-assimilated carbon in different species such as crops [24,25], trees [26-28] and grasses [29,30]. In most of these approaches, a setup containing polyethylene bags or chambers mounted over single leaves yielded valuable information of carbon allocation. In Arabidopsis, however, ^14^C feeding was used only rarely, which is surprising when considering the importance of this model species in plant biology today. When labelling was performed with Arabidopsis, it was generally on whole plants [31-34] or detached leaves [35,36]. Due to its very small, narrow leaves and rosette growth habit, Arabidopsis needs a special cuvette design for single leaf labelling experiments to avoid damage to the plant and to prevent contact of the isotopic label to other tissues.

In the present work, we introduce a cuvette for isotopic labelling of single leaves of intact Arabidopsis plants. The cuvette can be used for short-term isotopic feeding experiments with different carbon isotopes. It is easy to handle, allowing rapid attachment/removal and therefore a medium- to- high throughput, which we demonstrate with ^14^CO_2_ labelling. We show that ^14^CO_2_ labelling gives reliable information of carbon transport into sink tissues, such as developing leaves and roots, and broad information about carbon partitioning into major metabolite classes and biosynthetic end-products. It has the advantage of being very sensitive, which allows for short labelling pulses. The processing of the plant material as well as the data analysis are fast and straightforward and can be easily adapted by any lab. Therefore, despite the new emerging techniques using ^11^C and ^13^C labelling, we argue that ^14^C labelling, still has much potential to help us understand carbon metabolism in plants.

## Results

### Design of the single leaf labelling chamber and control experiments

**Figure 1:**
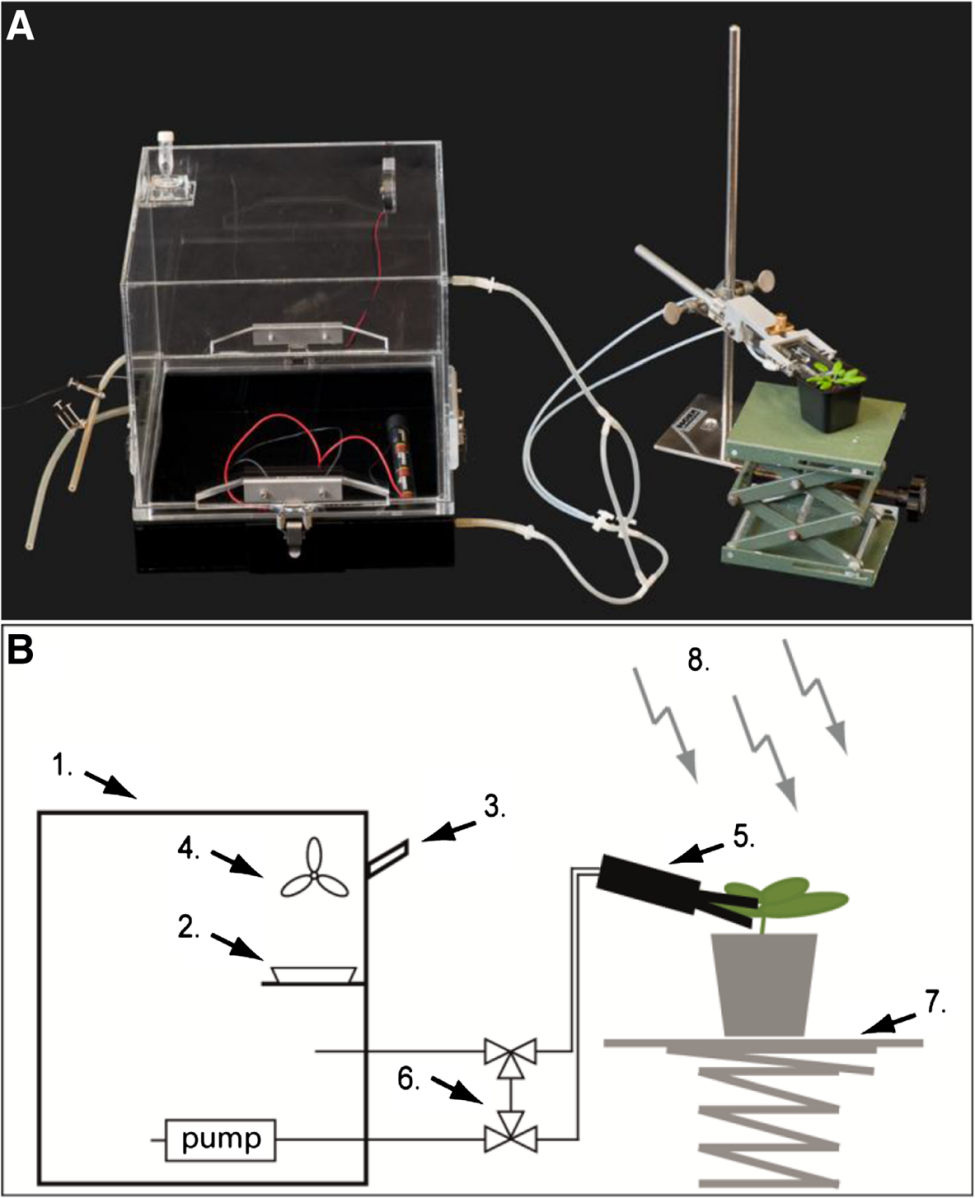
**Chamber for isotopic labelling of individual leaves. (A)** Picture of the single leaf labelling chamber. The labelling system comprises of a reservoir chamber (1) in which the isotopically labelled sodium-bicarbonate is placed within a petri dish (2). The bicarbonate is acidified by injecting an excess of lactic acid through a rubber seal (3) into the petri dish, releasing labelled CO_2_, which is homogenously dispersed within the chamber with a fan (4). The labelled air is pumped through a tubing system to the single leaf cuvette (5) or through a bypass (6) when exchanging leaves to be labelled. An adjustable table (7) is used to position the plants in relation to the external light source (8). **(B)** Scheme of the single leaf labelling chamber in use.

We designed equipment for isotopic labelling experiments of individual leaves in-house (Figure 1). The equipment comprises a sealed reservoir chamber, in which the isotopically labelled carbon is released, connected via tubing to the leaf cuvette, which can be clamped over a single leaf. Isotopically labelled gas is circulated from the reservoir via the tubing to the cuvette in order to supply the labelled carbon to an individual leaf. In the tubing system are two valves that allow or prevent airflow between the reservoir chamber and the labelling cuvette. These valves allow leaves to be inserted and removed from the cuvette without loss of labelled CO_2_ from the reservoir chamber.

**Figure 2:**
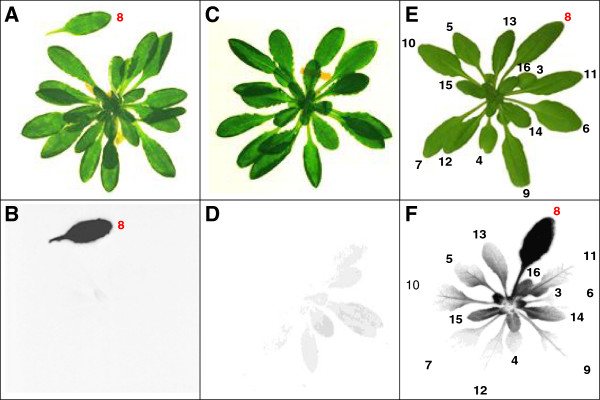
**Testing of the single leaf labelling cuvette.** Pictures **(A, C, E)** and autoradiograms **(B, D, F)** of Col-0 rosettes labelled with ^14^CO_2_. **(A and B)** Leaf 8 was introduced to the cuvette when still attached to the rosette. Just before labelling for 10 min with ^14^CO_2_, the petiole of leaf 8 was cut to prevent phloem export. **(C and D)** A rosette was placed for 60 min next to the leaf cuvette, which was being used to label other plants during a series of 5 pulse and chase experiments. **(E and F)** The ^14^C distribution pattern observed when the leaf cuvette is deliberately opened slightly during labelling in order to cause a leak. Leaf 8 was labelled for 5 min followed by a chase period of 1 h. The leaves are numbered according to the sequence of emergence where 1 is the first emerging true leaf after the cotyledons. Here, leaves 3 and upwards are indicated. Dried plant material was exposed to the film for 7 days.

We first tested the equipment for gas-tightness. Only a sealed system will allow the accurate analysis of transport processes from a labelled leaf to unlabelled tissues – a leak would result in inadvertent fixation of isotopically labelled carbon by the unlabelled tissues. For this test, a mature leaf (leaf 8) still attached to the rosette was clamped into the leaf cuvette. Just before opening the valves to allow the flow of ^14^CO_2_-containing air to the leaf cuvette, the petiole of the leaf was cut to prevent phloem export. After 10 min labelling, the rosette and the cut leaf were harvested, rapidly dried, and analysed by autoradiography (Figure 2A). The cut leaf gave a strong signal on the film, but hardly any signal from the rosette was visible (Figure 2B). The experiment was repeated and the labelled leaf and the unlabelled rosette were harvested separately. The amount of ^14^C incorporated into each part was determined by liquid scintillation counting. In the labelled leaf, around 800,000 disintegrations per minute (dpm) were detected, compared with 1,200 dpm in the unlabelled rosette. This is 0.15% of the overall label found in the plant. Thus, if the cuvette is properly attached to the leaf, the amounts of ^14^CO_2_ escaping from the chamber for assimilation by other leaves is negligible.

The autoradiographs and the quantitative measurements show that labelling for 10 min is ample and can be even reduced (e.g. to 2–5 min). The extent of carbon uptake from the chamber was calculated to ensure that the labelled CO_2_ is not depleted. The released radioactivity (300 μCi) corresponds to 666 million dpm. After 10 min labelling 801,200 dpm were detected in the whole plant, corresponding to 0.12% of the total label. Many plants can therefore be labelled successively without a significant reduction of the CO_2_ content in the reservoir.

The labelling of the leaves (the pulse) and the subsequent chase period were performed in the same fume hood under fluorescent lights. In practice, small amounts of ^14^CO_2_ inevitably escape during the experiment, either as replicate plants are exchanged into the cuvette or due to the respiration of labelled plants. To evaluate the amount of available ^14^CO_2_ within the fume hood, a second control experiment was performed in which a plant was positioned for 60 min next to the leaf cuvette while a series of 5 other plants were labelled for complete pulse and chase cycles. Subsequently, the unlabelled plant was harvested and analysed by autoradiography. The autoradiogram showed a faint signal for the leaves orientated towards the labelled plants (Figure 2C and D). The experiment was repeated and the rosette was harvested to quantify the ^14^C incorporated into the tissue, using liquid scintillation counting. The control plant beside the cuvette contained less than 0.1% of the ^14^C detected in the average labelled plants. Thus, there is negligible accumulation of ^14^C in the fume hood during the experiments and assimilation of free ^14^CO_2_ during the chase period will not significantly influence the labelling pattern (especially as the plants were not left adjacent to the leaf cuvette or the reservoir).

**Figure 3:**
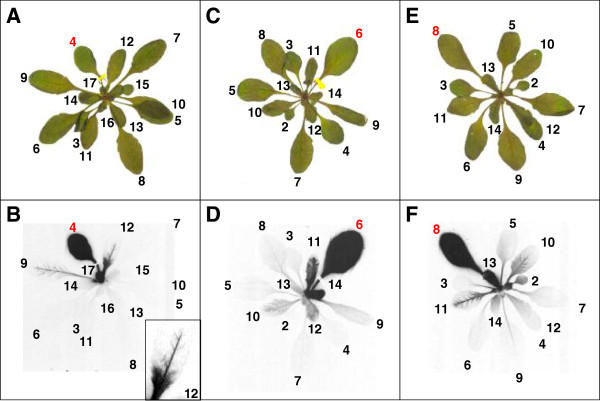
**Autoradiograms visualizing carbon transport within an Arabidopsis rosette.** Pictures **(A, C, E)** and autoradiograms **(B, D, F)** of Col-0 rosettes labelled with ^14^CO_2_. Leaf 4 **(A and B)**, 6 **(C and D)** and 8 **(E and F)** were labelled for 5 min followed by a chase period of 1 h. A close-up of leaf 12 is shown in B. The leaves are numbered as described in Figure 2. Plant material was dried and exposed to the film for 10 days.

**Figure 4:**
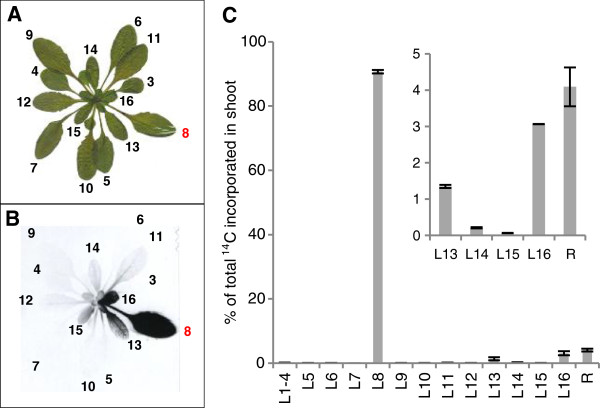
**Qualitative and quantitative representation of carbon export within an Arabidopsis rosette.** Picture **(A)** and autoradiogram **(B)** of a Col-0 rosette where leaf 8 was labelled with ^14^CO_2_, as described in Figure 2. The plant was exposed to the film for 9 days. **(C)** Quantitative analysis of the amount of ^14^C in different rosette leaves. Mean ± SE (n = 4). In this experiment, leaf 8 was labelled for 5 min, followed by a chase period of 1 h in air. The leaves were harvested separately for quantitative analysis. Leaf 1 (L1) is the first emerging leaf, L2 the next etc. R denotes the sample containing the leaves younger than leaf 16, the stem and meristem.

**Figure 5:**
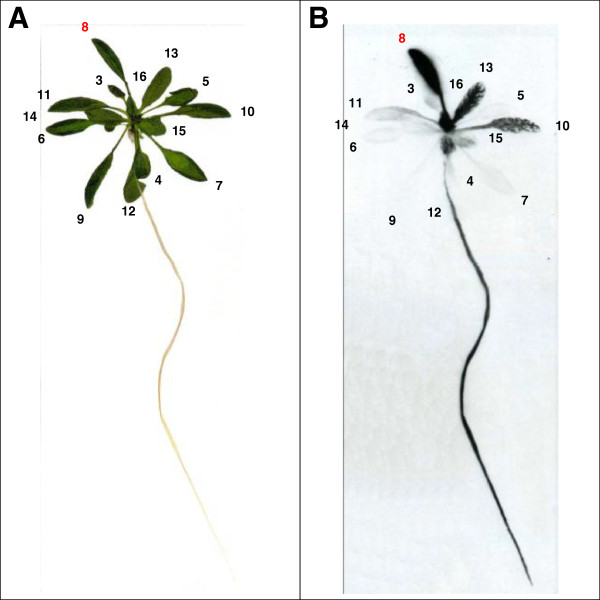
**Autoradiogram visualizing carbon transport to sink tissues.** Picture **(A)** and autoradiogram **(B)** of a Col-0 plant labelled with ^14^CO_2_. Leaf 8 was labelled for 5 min followed by a chase period of 1 h. The leaves are numbered as described in Figure 2. Plant material was exposed to the film for 13 days.

To demonstrate the ^14^C distribution pattern resulting from a non-gas-tight chamber, a leak was deliberately introduced while labelling a single leaf. A mature leaf (leaf 8) was labelled with ^14^CO_2_ for a 5-min pulse in a leaky chamber, followed by a chase period of 1 h. After the chase, the plants were analysed by autoradiography (Figure 2E and F). A strong signal was seen for the labelled leaf, but signals were also observed from many of the other leaves, both younger and older than leaf 8. The pattern of ^14^C was weaker from tissues that were furthest from the leaf cuvette, but otherwise not dependent on the position of the leaves on the rosette. This distribution of label represents a combination of export from the labelled leaves and assimilation of leaked ^14^CO_2_ by tissues adjacent to the cuvette and differed from the patterns derived with a sealed chamber (see Figures 3, 4 and 5).

### Analysing carbon transport processes with the single leaf labelling chamber

We used our system to illustrate the pattern of carbon allocation from source tissues to sink tissues in Arabidopsis qualitatively using autoradiograms and quantitatively by measuring the amount of incorporated radioactivity via liquid scintillation counting. The first experiment aimed to reveal if a defined pattern of carbon translocation can be seen in Arabidopsis. Different mature leaves were labelled with ^14^CO_2_ for 5 min, and the translocation of label into the rosette after a 1-h chase in air was analysed by autoradiography. Soil-grown, non-flowering plants with 17 to 19 leaves were used. Considering the first true emerging leaf as leaf 1, we chose leaves 4, 6 and 8 for labelling (Figure 3). All three labelled leaves showed a strong signal on the autoradiogram. Additionally, signals from other leaves were detectable. Labelling leaf 4 resulted in a strong signal in leaf 17 and weaker signals, mainly along the major veins, in leaves 9 and 12. In leaf 12, the base of the leaf was labelled strongly, but in the tip, only the major veins were labelled. When leaf 6 was labelled, a strong signal could be seen in leaves 14 and 11. Labelling leaf 8 led to a strong signal in leaves 13, 15 and 16, and accumulation of label along the major vein system in leaf 10 and 11. In each experiment, most of the other rosette leaves showed a weak background signal.

The export patterns in the three autoradiograms revealed that, in each case, carbon was predominantly exported to young, neighbouring leaves. Young leaves on the opposite side of the rosette did not import significant amounts of labelled carbon, nor did leaves older than the labelled leaf, irrespective of their rosette position. A similar pattern of carbon allocation has been described for several different species. The transport of carbon follows the vascular connections of the plant, which form along the orthostichies (i.e. between leaves that lie directly above each other; [19,37,38]). Very small developing leaves showed a strong signal evenly dispersed over the whole leaf blade, but larger developing leaves showed a signal mostly restricted to the veins. For several C-importing leaves an increase of signal towards the leaf base was seen. This gradient in carbon uptake is consistent with the idea that developing leaves undergo a basipetal sink-to-source transition, where the leaf tip matures first and stops importing carbon [15,39,40]. During this transition, the leaf tip exports carbon while the base still imports it. Gradually, as the leaf grows, there is a wave of maturation from tip to base until the whole leaf acts as a source tissue [41-43].

To quantify the carbon exported from a single leaf, leaf 8 was labelled as described above in several replicate plants. After the chase, the aerial parts of the plants were harvested for autoradiography (Figure 4A and B) or to quantify ^14^C in each leaf (Figure 4C). Of the total ^14^C incorporated during the pulse, 91% remained in the labelled leaf after the chase. Labelled carbon could also be detected in leaf 13 (1.3%), leaf 16 (3%) and the remaining part of the rosette containing all leaves smaller than leaf 16 and the shoot apex (4.1%). In all other harvested leaves, only 0.02% to 0.2% of the label could be measured, accounting for less than 0.8% of the assimilated carbon in total. These data confirmed the results gained from the autoradiograms (i.e. that most exported carbon was allocated to small developing leaves adjacent to the labelled leaf).

To monitor carbon translocation to the roots – another major carbon sink - plants were grown in hydroponic culture. Leaf 8 was labelled with ^14^CO_2_ as described above. Subsequently, autoradiograms were performed, revealing carbon transport on a whole plant level (Figure 5). The same pattern of carbon allocation could be seen as in the previous autoradiograms. Labelling leaf 8 leads to a strong signal in leaf 13 and leaf 16 and a signal in the veins of leaf 10. Additionally, the root gives a strong signal. This simultaneous transport towards the shoot apex and towards the roots of the plant was first reported decades ago [14,18,44]. However, it is still not known how the bidirectional transport is facilitated within the vascular system.

### Time course of carbon export to the root and the shoot

**Figure 6:**
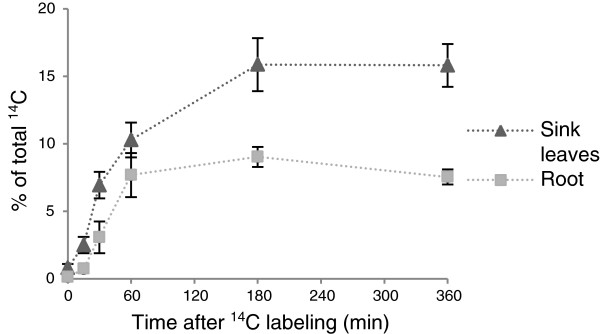
**Export of **^**14**^**C to the root and rosette leaves.** Leaf 8 of Col-0 plants was labelled for 5 min at 6 h into the light period with ^14^CO_2_, followed by chase periods of 0, 15, 30, 60, 180 or 360 min. At each time point, the labelled leaf, the unlabelled (sink) leaves and the root were harvested separately, and the incorporation of ^14^C was determined. The sum of the label in the labelled leaf, the sink leaves and the root is set to 100%. Mean ± SE (n = 5).

In the previous experiments, a 1-h chase was applied before analysing the plant tissue. To visualize the dynamics of carbon export from a source leaf into sink leaves and the root, time course experiments were carried out using hydroponically-grown plants. Leaf 8 (of plants with 16 to 20 leaves) was labelled with ^14^CO_2_ for 5 min in the middle of the light period, followed by a chase in air varying from 0 to 360 min. The labelled leaf, the unlabelled leaves and the root were harvested separately after each chase period and the ^14^C in each sample was determined (Figure 6).

Immediately after the pulse, export into the sink leaves was observed. One percent of the labelled carbon was detected in the unlabelled leaves. After a 15-min chase, 2.5% of the label was detected in the sink leaves. The amount of export increased rapidly during the first hour of the chase, after which the rate of export was slower, reaching a value of 16% after 180 min. The export values remained stable thereafter. Export of label to the roots was slightly delayed compared with export into rosette leaves. A significant amount of labelled carbon (3%) could only be detected after 30 min. The amount of imported carbon increased in the following 30 min up to 7.5% (after 1 h chase) and remained at around 7.5% - 9% thereafter. The release of CO_2_ from the plants was not measured during the chase period, but certainly labelled carbon was lost during the course of the experiment due to respiration, particularly from heterotrophic tissues like the root. This means that our values are slight underestimations of the exported amount of carbon. Zeeman and ap Rees [31] detected that 3% of the total label assimilated during a 1-h pulse was respired from wild-type roots during a five hour chase period.

### Time course of carbon partitioning into major metabolic pools

The previous experiments demonstrated the translocation of carbon from source to sinks. However, isotopic labelling can also be used to analyse the partitioning of carbon into the different major metabolite pools and biosynthetic end-products. Therefore, we fractionated our plant material into soluble and insoluble compounds after ^14^C labelling. Soluble compounds were further separated by ion-exchange chromatography into neutral, acidic and basic compounds. The neutral fraction is enriched in sugars, like sucrose, glucose and fructose. The acidic fraction is enriched in organic acids and sugar phosphates, and the basic fraction is enriched in amino acids. The insoluble fraction was further processed by specific enzymatic digestions, allowing the amount of carbon introduced into starch, proteins and other insoluble compounds (i.e. cell wall material) to be determined. The proportion of assimilated ^14^C in each fraction was measured using liquid scintillation counting. These data reveal the amount of assimilated carbon utilized in different biosynthetic pathways.

To assess carbon partitioning within source tissues, a time course experiment was performed using soil-grown plants. Leaf 8 was labelled for 5 min followed by chase periods between 0 and 180 min. At given times, the labelled leaves were harvested and the amount of carbon partitioned to the water-soluble compounds, ethanol-soluble compounds and insoluble compounds was determined (Table 1). After the pulse, most ^14^C was found in the water-soluble fraction, with the basic, acidic and neutral fractions all containing significant amounts of label. The amount of ^14^C in acidic and basic compounds decreased markedly after the 15-min chase, indicative of a rapid turnover of the metabolic intermediates. A fast incorporation of carbon and high turnover rate can be expected for intermediates of the Calvin-Benson cycle (found in the acidic fraction) and for photorespiratory intermediates (such as serine and glycine in the basic fraction). Label in the basic compounds continued to decrease after the initial rapid drop during the first 15 min of the chase period. From the 25% of label just after the pulse, only 5% remained in this pool after a 180-min chase period. Some amino acids may be used for protein synthesis and an increase in label in protein was observed during the time course. However, most of the label is probably cycled into other pools as photorespiration and photosynthesis continue throughout the chase. Consistent with this, after the rapid decrease of label in the acidic fraction from 0 to 15 min chase (most likely representing turnover of sugar phosphates from the Calvin-Benson cycle), the amount of label increased again from 14% to 23% after 180 min chase. This increase probably represents the synthesis of organic acids (e.g. malate and fumarate) that are stored in the vacuole. A peak of labelled carbon incorporated into the neutral fraction appeared after the 15 min chase. The amount of label decreased thereafter from 22% to 7%. The peak after 15 min most likely represents labelled sucrose, which is then either metabolized within the tissue or exported to sink tissues.

**Table 1:** Time course of carbon partitioning into the major carbon compound classes in the labelled leaf

			**% of **^**14**^**C recovered in each fraction related to the total amount per tissue**				
			**After pulse**	**15 min chase**	**30 min chase**	**60 min chase**	**180 min chase**
**Labeled leaf**^**1**^			**100**	**100**	**100**	**100**	**100**
	**Soluble**		**77.0 ± 2.2**	**58.4 ± 3.5**	**59.3 ± 2.1**	**46.2 ± 3.4**	**44.7 ± 2.4**
		Neutral	14.6 ± 0.9	23.1 ± 1.2	22.8 ± 1.9	11.6 ± 2.0	7.3 ± 0.9
		Acidic	27.7 ± 0.9	15.1 ± 0.7	17.0 ± 1.8	20.0 ± 1.5	25.9 ± 1.8
		Basic	24.9 ± 1.4	14.3 ± 1.2	11.3 ± 1.3	8.2 ± 0.5	5.4 ± 0.5
	**Insoluble**		**17.3 ± 2.0**	**30.5 ± 4.3**	**36.6 ± 1.9**	**42.5 ± 4.6**	**45.1 ± 4.7**
		Starch	16.6 ± 1.1	27.7 ± 2.4	29.3 ± 2.2	34.4 ± 3.1	37.2 ± 3.2
		Protein	0.8 ± 0.2	3.1 ± 0.7	3.1 ± 0.5	4.6 ± 0.5	4.2 ± 0.5
		Others	0.3 ± 0.1	2.0 ± 0.6	2.3 ± 0.3	3.8 ± 0.2	3.7 ± 0.4
	**EtOH soluble**		**1.7 ± 0.1**	**4.0 ± 0.6**	**4.1 ± 0.4**	**5.6 ± 0.2**	**5.7 ± 0.3**

^1^Leaf 8 of Col-0 plants was labelled for 5 min with ^14^CO_2_, 6 h into the light, followed by a chase period of 0, 15, 30, 60 or 180 min. After the chase, the labelled leaf was harvested and the incorporation of ^14^C was determined. The sum of the label in the labelled leaf is set to 100%. The relative amount of ^14^C incorporated into the different compound classes (as a percentage of the total label in the leaf) is shown. Mean ± SE (n ≥ 5).

While 80% of the label was found in the water-soluble fraction right after the labelling pulse, 17% were found in insoluble compounds (mainly starch). Carbon was very rapidly partitioned into starch, as half of the label detected in starch after 180 min chase was already found in this pool after the 5-min pulse. The amount of label in starch increased from 17% to 27% after the 15-min chase period, followed by a further increase to 37% over the rest of the chase. In the other insoluble compounds, like proteins and cell wall material, hardly any label could be detected immediately after the labelling experiment, but the amount of label increased over the first hour of the chase. After a further two hours chase, only minor changes were seen. Carbon partitioning into ethanol-soluble compounds (representing lipids, pigments and waxes) increased from 1.7% immediately after labelling to 5.1% after 1-h chase. No more ^14^C was introduced into the ethanol-soluble compounds in the subsequent two hours. The pattern is similar to that of other end-products like proteins and cell wall material. This reveals that approximately half of the assimilated carbon is either used immediately for storage (in the form of starch) or it is channelled to its final destination within the first hour after fixation. For the longer chase periods, it should be noted that the percentage values might be slight overestimates, as carbon export into the roots was not monitored in this experiment. As carbon is exported during the chase, less label is retained in the rosette, so the percentage of ^14^C in the fractions of the labelled leaf increases even if the absolute amount of ^14^C is constant.

## Discussion

Here, we have shown that our labelling chambers are air-tight, fast to attach and to remove, and therefore ideal for labelling experiments with single Arabidopsis leaves. During the experimental procedure only minimal amounts of labelled CO_2_ were released from the system, probably while attaching and removing the chamber, causing negligible incorporation of ^14^C by unlabelled leaves.

Feeding labelled carbon to individual leaves of intact plants has the advantage that carbon transport and carbon partitioning (in the specific source tissue) can be monitored simultaneously. It allowed us to determine, qualitatively as well as quantitatively, the amount of assimilated carbon which is transported form source leaves to sink leaves and other sink tissue such as the root in parallel. A defined pattern of carbon allocation from a mature leaf could be seen. A similar pattern, which is determined by the vascular system, was observed for other plants like tobacco [18,19]. Following the vascular connections, carbon is transported from a mature leaf upwards to the leaves n + 3, n + 5, n + 8 and n + 10 in tobacco plants. For Arabidopsis, two vascular models have been described which either suggest connections from one leaf to the leaves n + 2 and n + 3 (from leaf 4 onwards; [45]) or to the leaves n + 5 and n + 8 [46]. In the majority of autoradiograms performed, we could see carbon import into leaves n + 5, n + 8 and, if already emerged, in leaf n + 13 (which is linked to the fed leaf *via* leaf n + 5 and n + 8). This supports the vascular model proposed by Kang et al. [46]. However, we occasionally observed carbon import into the leaves n + 2 and n + 3 (Figures 3, 4 and 5) which could be explained by stochastic irregularities during the formation of vascular connections.

A simultaneous transport from mature leaves towards developing leaves and towards the root was seen in the autoradiographs as well as in the quantitative measurements. It suggests that, in certain regions of the vasculature, bidirectional phloem transport occurs. Bidirectional transport within the phloem is long-known, but it is still unclear how it occurs [14,18,44]. According to our current understanding of the phloem, simultaneous transport within one sieve tube cannot occur. Therefore, a more complex phloem structure with separate cell files facilitating acropetal and basipetal transport seems likely [47,48]. The labelling system presented in this work could help gain new insights into carbon transport and a better understanding of phloem structure/function.

The quantitative export data revealed that about 25% of the assimilated carbon is exported from the labelled source leaves within the same photoperiod, most of it already within the first hour after fixation. Higher export rates were previously described for sugar beet, where 24% of carbon was exported to sink leaves after a 1-h chase period [16]. In *Curcurbita pepo*, 50% of label from a single leaf was found to be exported after a 2-h chase period, with 16% allocated to the roots [15,49]. Export processes in the dark have not been measured in this study, but are likely to be significant. Experiments with whole Arabidopsis rosettes by Zeeman and ap Rees [31], using similar conditions to those described here (i.e. labelling in the middle of the photoperiod), revealed 6.9% export to the root by the end of the day, comparable to our results. However, by the end of the subsequent night, export from the rosette to the root had increased to 22.7%. This night-time export is driven by carbon released from starch during the night, significantly increasing the amount of carbon allocated to sinks during the whole day-night cycle. The time course analysis of the labelled leaf also showed that, as well as storage compounds like starch, carbon flows via intermediary metabolite pools towards the synthesis of end products like cell wall and proteins. These data suggest that leaf 8, despite being one of the most mature leaves on the rosette, is still growing, so not all assimilated carbon will be destined for export. This is consistent with sensitive growth analysis data which reveal a continuation of Arabidopsis leaf expansion for up to 3 weeks [50].

While the flow through acidic and basic fractions was very rapid after the labelling pulse, carbon flow through neutral compounds (e.g. sucrose, glucose and fructose) was slower and peaked well after the end of the labelling - between 15 to 30 min into the chase. Carbon incorporation into starch was fast, with the majority of carbon incorporation during the pulse period and the following 15-min chase. These data are broadly in line with data recently obtained in a detailed study using ^13^C labelling [51], where the degree of label saturation in the different metabolic intermediates followed quite different kinetics during one hour of labelling (presumably depending on both fluxes and intermediate pool sizes). In that study, sucrose labelling was relatively slow, with glucose and fructose labelling slower still. However, ADPglucose - the precursor for starch biosynthesis - was rapidly labelled. These data are also consistent with the dynamics we observed in label export from the leaf, which approached completion an hour after the pulse. It should be emphasized that, compared with the fairly crude separation of compound classes presented here, ^13^C labelling coupled with mass spectrometry gives excellent resolution in terms of the degree of labelling of intermediates over time. However, even with today’s technology it is still a major undertaking to measure metabolite fluxes with ^13^C time-course labelling.

## Conclusions

We have shown the design and functionality of a system for single-leaf isotopic labelling in Arabidopsis suitable for both ^13^CO_2_ and ^14^CO_2_. Although not widely used in the field of plant physiology, ^14^C-labeling remains a valuable tool. The method is inexpensive and can be introduced easily in the laboratory. Moreover, our study shows that short labelling times are sufficient to obtain robust data, allowing for a medium to high throughput. The method is ideal for measuring carbon export into sink regions, as we recently demonstrated for a mutant affected in callose synthesis in the phloem [52], as well as carbon partitioning within tissues. With sufficient accumulation of radioactivity in the sample, partitioning of fixed carbon in the labelled tissues, as well as imported carbon in unlabelled tissues, can be analysed in further detail. For example, it would be possible to analyse partitioning of assimilated carbon as well as imported carbon in a growing leaf. It would also be possible to study carbon partitioning in response to changing environmental conditions or to observe altered partitioning patterns in any of the vast number of Arabidopsis mutant lines available today. Thus, we suggest that by giving insight into whole-plant carbon allocation, ^14^CO_2_ feeding experiments can help to elucidate how carbon metabolism is controlled and provide valuable data for models of plant growth and biomass production.

## Methods

### Isotopic labelling chambers

A reservoir chamber and a single leaf labelling cuvette were designed and constructed in-house. All chambers are made of Plexiglas with EPDM foam rubber to seal between the different components. The reservoir chamber comprises a lower part made of black Plexiglas, a lid made of transparent Plexiglas, and has an internal volume of 17.3 litres. The chamber is closed via four clamps, sealing with a foam gasket between the upper and lower parts. In the lid, a holder and a fan are mounted. A petri dish containing the isotopically labelled sodium bicarbonate is placed on the holder and acidified with lactic acid to liberate isotopically labelled CO_2_, which is distributed by the fan. The chamber has two inlets and two outlets which can be used to connect it to other devices (i.e. the leaf cuvette), or sealed if not used. The leaf cuvette is 7 cm long and 3 cm wide and was constructed to label individual leaves of an Arabidopsis plant (see Figure 1). With the leaf cuvette, leaves with a minimum length of approximately 2 cm and a maximum length of 6.5 cm can be labelled. The cuvette consists of a lower and an upper part made of transparent Plexiglas which can be clamped over single leaves. The Plexiglas parts are surrounded by foam gasket to enable an air-tight sealing, yet avoid damage of the leaf petiole. In the lower part of the cuvette is an inlet and an outlet for connecting it to the reservoir chamber containing the isotopically labelled gas. The inlet and outlet feed into opposite ends of the leaf cuvette. A small battery driven pump (Nitto Kohki, Germany) within the reservoir chamber provides an even air-flow (250 ml/min) through the leaf cuvette (Figure 1). Labelling times of 5 to 10 min were typically used, meaning that many replicate plants can be labelled within one experiment. A bypass with three-way taps allows the cuvette to be isolated from the reservoir chamber while replicate plants are exchanged. Further design information on the apparatus is available on request. The entire apparatus was placed inside a fume hood, illuminated with fluorescent bulbs similar to those in the growth chambers used for plant cultivation.

### Plant growth conditions

For soil grown plants, *Arabidopsis thaliana* seeds were sown on Einheitserde Typ ED 73. Sown seeds were stratified for three days at 4°C before being transferred to a Percival AR95 growth cabinet with a 12-h photoperiod (light intensity, 150 μmol quanta m^-2^ s^-1^; temperature, 20°C; relative humidity, 65%). For hydroponic culture, seeds were sown in 1.5-ml microcentrifuge tubes filled with 0.65% (w/v) plant agar. The bottom of the tubes was cut off and the tubes were placed in a rack with the cut ends immersed in nutrient solution [53]. The seeds were stratified grown as specified above. The nutrient solution was renewed weekly.

### ^14^CO_2_ pulse-chase labelling

Labelling experiments on single leaves were performed using the same light conditions specified above. By acidification of sodium ^14^C-bicarbonate (Hartmann Analytic GmbH, Germany; supplied with a specific activity of 59.5 mCi/mmol), ^14^CO_2_ was released in the sealed reservoir chamber. This increased the CO_2_ content in the chamber by only 5–10 ppm. The leaf cuvette was clamped onto an individual mature leaf of the plant. After a ‘pulse’ period (as specified, but typically 5 to 10 min), the cuvette was isolated from the reservoir, opened, the leaf removed, and the plant kept in normal air for a chase period (as specified, but typically 60 min).

### Autoradiography

To visualize transport of carbon within the Arabidopsis rosette, autoradiograms were taken after ^14^CO_2_ labelling of single leaves. Following the chase period, either the rosette was removed from the root (soil-grown plants) or the whole plant was taken (hydroponically-grown plants) and placed between four layers of filter paper. The plant material was immediately dried at 80°C in a gel drier for 20 min. After drying, the filter paper was exchanged and the plants were further flattened under a weight for 1 week. Afterwards, the plant material was wrapped in transparent cling film and exposed to a Kodak BioMax MR film (as indicated, but typically for 1 week).

### Extraction and fractionation of ^14^CO_2_ labelled plant tissue

Liquid scintillation counting was used to determine the ^14^C incorporated into different metabolic fractions. Plant material was harvested and quenched in boiling 80% (v/v) ethanol. The tissue was homogenized using an all-glass homogenizer and extracted in successive 10 min incubations in 80% (v/v) ethanol, 50% (v/v) ethanol, 20% (v/v) ethanol, water, and finally 80% (v/v) ethanol. Between each extraction step the insoluble cell debris was removed by centrifugation. The supernatants were combined and dried *in vacuo*. The dry residue was dissolved in 2 ml water, giving the water-soluble fraction. Any water-insoluble residue was dissolved in 1 ml 98% (v/v) ethanol and referred to as the ethanol-soluble fraction. The insoluble cell debris was resuspended in 1 ml water. The total ^14^C in the insoluble fraction was determined by dissolving an aliquot in tissue solubilizer (NCS, GE Healthcare) for 16 h at 23°C. The sum of the label detected in the water-soluble, the ethanol-soluble and the insoluble fractions from all parts of the plant gave the total incorporated ^14^C. The sum of the label in the unlabelled leaves and the root (where analysed) gave the amount of ^14^C exported from the labelled leaf.

To measure the ^14^C incorporated into starch, the insoluble material was digested with amyloglucosidase and α-amylase (from *Aspergillus niger* and pig pancreas, respectively; Roche) for 16 h at 37°C. The incorporation of ^14^C into proteins was determined by protease digestion (Protease Type XIV from *Streptomyces griseus*, Sigma-Aldrich) for 16 h at 37°C. The amount of ^14^C in the remaining insoluble material (primarily cell wall material) was determined by subtracting the ^14^C in starch and protein from the overall ^14^C in the insoluble fraction. The water-soluble fraction was further separated into neutral, acidic, and basic compounds by ion exchange chromatography on sequential columns of a cation exchanger (Dowex 50WX4) and an anion exchanger (Dowex1X8) as described in Zeeman and ap Rees [21]. The resulting neutral fraction was enriched for sugars such as sucrose, glucose and fructose, the acidic fraction was enriched for organic acids and sugar phosphates, and the basic fraction was enriched for amino acids.

The incorporation of ^14^C into the different metabolic pools was determined by measuring an aliquot of the respective fraction via liquid scintillation counting. Therefore, the aliquot was adjusted to 1 ml with water and 4 ml scintillation cocktail were added. For the ethanol, starch and acidic fractions, Ultima Gold LLT Scintillation Cocktail was used, for all other fractions Ultima Gold Scintillation Cocktail was used (both from Perkin Elmer, Switzerland). After an incubation period of 30 min in the dark, the sample was measured with a LS1801 scintillation counter (Beckmann, Switzerland).

## Competing interests

The authors declare that they have no competing interests.

## Authors’ contributions

SZ and KK conceived the project. SZ, KK and PF designed and built the apparatus. KK and AM carried out the experiments. KK and SZ drafted the manuscript. All authors read and approved the final manuscript.
